# Handheld ultrasound-assisted versus palpation-guided combined spinal-epidural for labor analgesia: a randomized controlled trial

**DOI:** 10.1038/s41598-023-50407-7

**Published:** 2023-12-27

**Authors:** Jinyoung Bae, Youngwon Kim, Seokha Yoo, Jin-Tae Kim, Sun-Kyung Park

**Affiliations:** 1 Department of Anesthesiology and Pain Medicine, Ajou University Medical Center, Ajou University School of Medicine, Suwon, Republic of Korea; 2 Department of Anesthesiology and Pain Medicine and Anesthesia and Pain Research Institute, Yonsei University College of Medicine, Seoul, Republic of Korea; 3 Department of Anesthesiology and Pain Medicine, Seoul National University Hospital, Seoul National University College of Medicine, Seoul, Republic of Korea

**Keywords:** Randomized controlled trials, Anatomy, Health care, Medical research

## Abstract

Preprocedural ultrasound assistance can enhance the efficacy of neuraxial anesthesia in obstetrics. We investigated whether the use of handheld ultrasound can shorten the procedural time of labor combined spinal-epidural (CSE) analgesia compared with conventional landmark-guided methods. Eighty-four women requesting labor analgesia were randomly assigned to either handheld ultrasound-assisted or palpation-guided CSE analgesia. Primary outcome was procedure time of the CSE analgesia. Secondary outcomes included identification time, performance time, number of needle manipulations required for epidural/spinal success, first-attempt success rate, periprocedural pain scores, the incidence of accidental dural puncture, and patient satisfaction. Total procedure time did not significantly differ between the ultrasound and palpation groups (median [IQR], 191.5 [167–224] vs. 204.5 [163–358] s; *P* = 0.442). However, the performance time was significantly shorter in the ultrasound group (134.5 [115–177] vs. 183 [129–296] s; *P* = 0.011), although identification time was longer in the ultrasound group (53 [41–72] vs. 30.5 [21–45] s; *P* < 0.001). The epidural success rate at first insertion attempt was higher in the ultrasound group (85.7% vs. 59.5%, *P* = 0.014). Preprocedural handheld ultrasound assistance resulted in equivalent total procedure times but reduced performance times and higher first-attempt success rates. Therefore, clinicians may consider this technique for labor CSE analgesia.

Trial registration: NCT04759547.

## Introduction

Neuraxial analgesia is the most effective method for labor pain relief in contemporary obstetric anesthesia^1–3^. Combined spinal-epidural (CSE) analgesia has a faster onset, better sacral coverage, and less use of rescue analgesia compared to simple epidural analgesia^2,4–6^. The most popular technique for CSE analgesia is the needle-through-needle (NTN) technique, in which the epidural needle is sited in the epidural space, and the spinal needle is introduced through the epidural needle to puncture the dura^7,8^. However, the spinal component of the technique may fail when the epidural needle is angled away from the midline, even if the epidural needle tip is correctly placed in the epidural space^4,8,9^.

Preprocedural ultrasound has been introduced as a useful tool for neuraxial anesthesia^10–14^. It can potentially aid the successful placement of CSE by providing anatomical information such as the depth of the epidural space and location of the neuraxial midline^10,11^. While ultrasound has been recommended for epidural placement in obstetrics^15^, its use for neuraxial block in the obstetric unit remains limited because of the lack of a dedicated ultrasound machine or concerns about the time it takes^13,16–18^. In most cases, labor neuraxial procedures are performed on demand and in a maternity unit separate from the main operating theater in many hospitals. In such clinical circumstances, a handheld ultrasound can be particularly useful. However, to date, no clinical trial has compared the use of handheld ultrasound assistance with conventional landmark palpation in laboring women receiving CSE analgesia. Furthermore, it remains undetermined whether the use of handheld ultrasound could enhance the efficacy of the CSE procedure in the general obstetric population.

Therefore, this study aimed to compare the efficacy between the preprocedural handheld ultrasound-assisted technique and the conventional landmark-guided technique in parturients undergoing CSE analgesia. We hypothesized that the handheld ultrasound-assisted technique would shorten the procedural time of labor CSE analgesia compared to the conventional technique.

## Results

**Figure 1:**
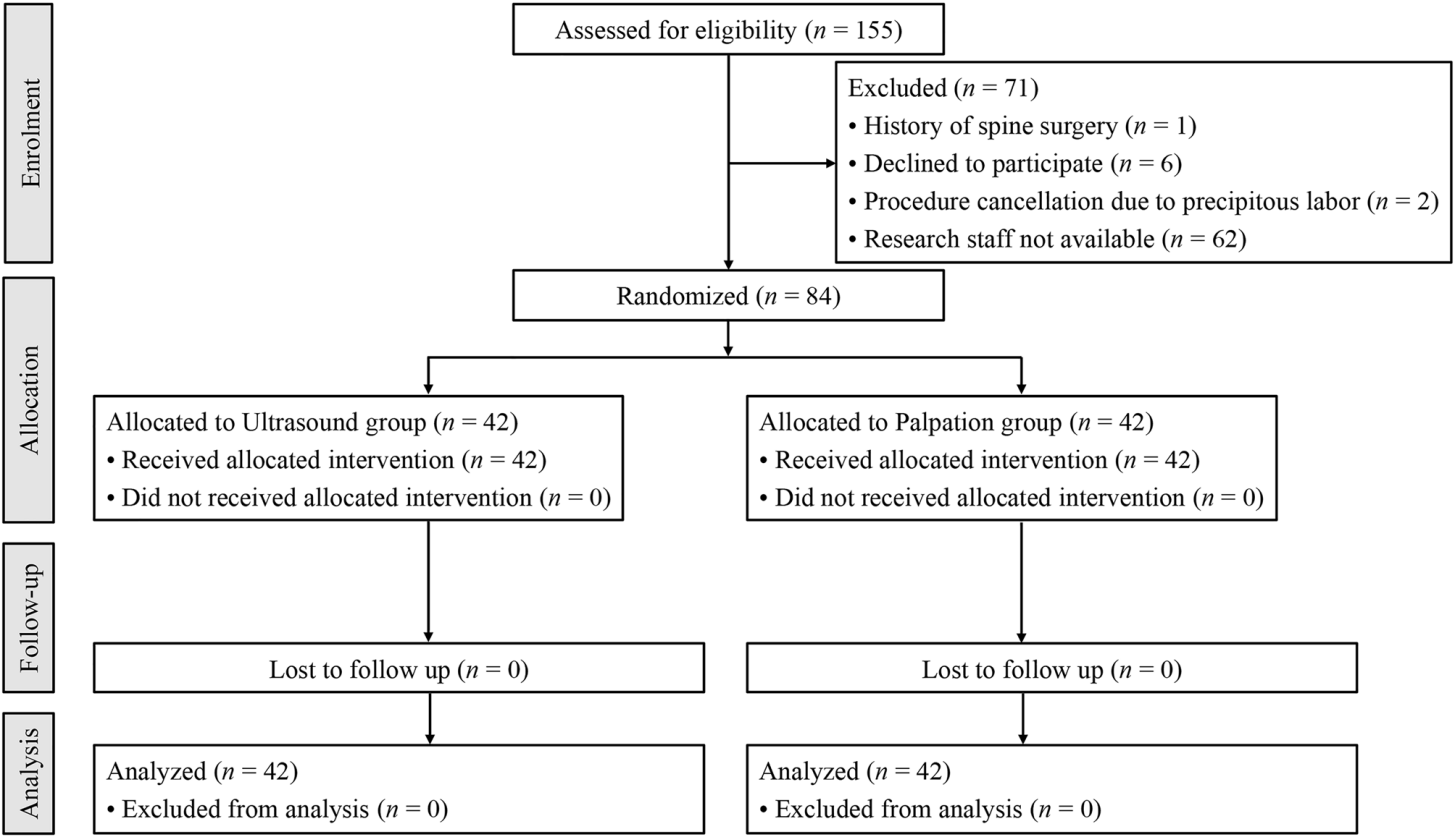
CONSORT flow diagram.

A total of 155 mothers were assessed for eligibility, and 84 were included in the final analysis (Fig. 1). Baseline characteristics were reported in Table 1. The median body mass index was 26.6 (IQR, 24.7–29.0 [range, 20.1–37.9]) kg/m^2^ for all patients. The percentage of mothers with a body mass index > 30 kg/m^2^ was 21.4% (9/42) in the ultrasound group and 11.9% (5/42) in the palpation group, respectively (Table 1).

**Table 1:** Baseline characteristics and procedural characteristics.

	Ultrasound group (n = 42)	Palpation group (n = 42)	*P* value
Age (y)	33.5 (32–38)	35 (32–37)	0.726
Height (cm)	162.7 (5.8)	161.8 (5.7)	0.472
Weight (kg)	72.9 (64.2–79.9)	68.5 (63.1–76.0)	0.225
Body-mass index (kg/m^2^)	27.1 (25.0–29.2)	26.3 (24.5–28.7)	0.320
Body-mass index > 30 kg/m^2^	9 (21.4%)	5 (11.9%)	0.380
Gestational age (weeks)	38 (37–39)	38 (37–40)	0.310
Nulliparous	31 (73.8%)	31 (73.8%)	1.000
Singleton pregnancy	33 (78.6%)	31 (73.8%)	0.798
Labor pain before CSE analgesia (NRS)	8 (7–10)	9 (7.5–10)	0.237
Ease of landmark palpation			0.920
Easy	19 (47.5%)	22 (53.7%)	
Moderate	12 (30%)	12 (29.3%)	
Difficult	8 (20%)	6 (14.6%)	
Impossible	1 (2.5%)	1 (2.4%)	
Performer of CSE procedure, A/B/C	14 (33%)/14 (33%)/14 (33%)	14 (33%)/14 (33%)/14 (33%)	1.000
Interspace level at which dural puncture was done			0.909
L3–4	21 (50%)	22 (53.7%)	
L4–5	21 (50%)	19 (46.3%)	
Epidural depth (cm)			
Needle depth at loss of resistance	5.0 (4.2–5.5)	5.0 (4.0–5.2)	0.512
Measured by handheld ultrasound	4.5 (4.0–5.3)	–	
Spinal needle depth at which CSF efflux was obtained (cm)^a^	5.5 (4.8–6.1)	5.4 (5.0–6.0)	0.703

Values are mean (SD) or median (IQR) or number (percentage). *CSE* combined spinal-epidural, *NRS* numeric rating scale, *CSF* cerebrospinal fluid.

^a^Spinal needle depth was measured using the scale marked on the CSE needle (CSEcure, Portex Combined Spinal/Epidural minipack 27G/18G, ICU Medical, Inc., San Clemente, CA).

The procedure time, defined as the sum of the identification time and performance time, showed no significant difference between the ultrasound and palpation groups (median [IQR], 191.5 [167–224] vs. 204.5 [163–358] s; difference of medians [95% CI], − 13 [− 86.5 to 23.5]; *P* = 0.442; Table 2). However, the performance time, defined as the time interval from epidural needle insertion to the end of application of the occlusive dressing, was shorter in the ultrasound group (134.5 [115–177] vs. 183 [129–296] s, *P* = 0.011). The identification time, defined as the time interval from the placement of ultrasound to the completion of skin marking in the ultrasound group or from the first touch for palpation to completion of palpation in the palpation group, was longer in the ultrasound group (53 [41–72] vs. 30.5 [21–45] s, *P* < 0.001). The total time including time taken for sterilization and local anesthetic infiltration did not significantly differ between the two groups (Table 2). We observed no significant difference in the procedure time between the three anesthesiologists (Supplemental Table S1).

**Table 2:** Procedural time of combined spinal-epidural anesthesia.

	Ultrasound group (n = 42)	Palpation group (n = 42)	*P* value	Difference of medians (95% CI)
Procedure time (s)^a^	191.5 (167–224 [135–598])	204.5 (163–358 [115–1260])	0.442	− 13 (− 86.5 to 23.5)
Identification time (s)^b^	53 (41–72 [20–115])	30.5 (21–45 [14–119])	< 0.001	22.5 (13.5 to 31.0)
Performance time (s)^c^	134.5 (115–177 [78–559])	183 (129–296 [99–1210])	0.011	− 48.5 (− 114 to − 4)
Time from epidural needle insertion to LOR (s)	40.5 (28–66 [10–130])	42 (30–89 [14–385])	0.279	− 1.5 (− 20.5 to 15.0)
Time from LOR to efflux of CSF (s)	15 (12–21 [7–394])	19 (16–30 [11–344])	0.011	− 4 (− 6.5 to − 0.5)
Time from CSF efflux to spinal needle removal (s)	29 (23–40 [13–69])	30.5 (25.5–38 [15–65])	0.626	− 1.5 (− 7.5 to 4.0)
Time from spinal needle removal to Tuohy needle removal (s)	40 (33–46 [18–157])	41 (37–53 [22–803])	0.128	− 1.0 (− 8.0 to 3.5)
Total time including preparation time (s)^d^	452.5 (412.5–511.5 [327–824])	492.5 (391.5–605.5 [336–1481])	0.567	− 40.0 (− 94.8 to 44.2)

Values are median (IQR [range]). *LOR* loss of resistance, *CSF* cerebrospinal fluid.

^a^Procedure time was defined as the sum of identification time and performance time.

^b^Identification time was defined as the time from the placement of ultrasound on the patient’s skin and the anesthesiologist’s declaration of completion of skin marking in the ultrasound group and the time from the first touch for palpation to completion of palpation in the palpation group.

^c^Performance time was defined as the time from epidural needle insertion to the end of application of the occlusive dressing of the epidural catheter.

^d^Total time including preparation time was defined as the time from the start of identification using ultrasound or palpation to the end of the application of the dressing.

The epidural catheter was successfully inserted in all the patients (Table 3). The NTN technique was successful in every mother in the ultrasound group. The first-attempt success rate of the epidural component was higher in the ultrasound group (85.7% vs. 59.5%; relative risk [95% CI], 1.4 [1.1–1.9]; *P* = 0.014; Table 3). However, the first-pass success rate of the spinal component without epidural needle redirection, the number of redirections until dural puncture, and the need for alternative methods did not significantly differ between the groups (Table 3). The results of the subgroup analysis of mothers with a body mass index > 30 kg/m^2^ are presented in Supplemental Table S2.

**Table 3:** Efficacy outcomes and periprocedural pain/discomfort scores.

	Ultrasound group (n = 42)	Palpation group (n = 42)	*P* value	Relative risk or median difference (95% CI)
Epidural success rate				
Overall	42 (100%)	42 (100%)		1 (1–1)
At first skin puncture attempt	36 (85.7%)	25 (59.5%)	0.014	1.4 (1.1–1.9)
At first needle pass	16 (38.1%)	18 (42.9%)	0.824	0.9 (0.5–1.5)
Needle-through-needle spinal success rate				
Overall	42 (100%)	40 (95.2%)	0.474	1.05 (0.98–1.12)
At first spinal needle pass	38 (90.5%)	33 (78.6%)	0.228	1.2 (0.9–1.4)
Number of epidural needle insertion attempts^a^	1 (1–1 [1–4])	1 (1–2 [1–4])	0.015	0 (− 1 to 0)
Number of needle passes^b^	2 (1–2 [1–15])	2.5 (1–6 [1–15])	0.190	− 0.5 (− 2 to 1)
Number of redirections of epidural needle for needle-through-needle spinal success			0.443	
0	38 (90.5%)	33 (78.6%)		
1	1 (2.4%)	2 (4.8%)		
2	2 (4.8%)	6 (14.3%)		
≥ 3	1 (2.4%)	1 (2.4%)		
Number of interspace levels at which the insertion was attempted			0.228	
1	38 (90.5%)	33 (78.6%)		
2	4 (9.5%)	9 (21.4%)		
Use of alternative methods	2 (4.8%)	3 (7.1%)	> 0.999	0.7 (0.1–3.8)
Periprocedural pain score (NRS)	2 (1–3 [0–5])	2 (1–4 [0–7])	0.419	0 (− 1.25 to 1.0)
Periprocedural patient discomfort score (NRS)	2 (1–3 [0–7])	3 (1–5 [0–9])	0.069	− 1 (− 2 to 0)

Values are median (IQR [range]) or number (percentage). *NRS* numeric rating scale.

^a^Number of epidural needle insertion attempts was defined as the number of individual needle skin punctures until the successful combined spinal-epidural placement.

^b^Number of needle passes was defined as the number of needle redirections without removing the needle from the skin until the first successful epidural placement.

No significant intergroup differences were found in the incidence of radicular pain, paresthesia, and bloody tap during the procedure (Table 4). Unintentional dural puncture with an epidural needle occurred in five patients in the palpation group but not in the ultrasound group (*P* = 0.065; Table 4). Failure of labor analgesia, post-dural puncture headache, and patient satisfaction with the procedure were not significantly different between the groups (Table 4).

**Table 4:** Analgesic outcomes, complications, and patient satisfaction.

	Ultrasound group (n = 42)	Palpation group (n = 42)	*P* value	Relative Risk or Median Difference (95% CI)
Labor pain 10 min after CSE procedure (NRS)^a^	1 [0–2]	0 [0–2]	0.097	1 (0 to 1.5)
Average labor pain after CSE analgesia (NRS)^b^	2 [0.5–3]	1.2 [0–4.5]	0.955	0.8 (− 1.8 to 1)
Failure of labor analgesia^c^	0 (0%)	2 (4.8%)	0.474	NA
Complications during CSE				
Radicular pain	5 (11.9%)	4 (9.5%)	> 0.999	1.3 (0.4–4.3)
Paresthesia	0 (0%)	1 (2.4%)	> 0.999	NA
Bloody tapping	2 (4.8%)	1 (2.4%)	> 0.999	2 (0.2–21.2)
Accidental dural puncture with epidural needle	0 (0%)	5 (11.9%)	0.065	NA
Periprocedural composite complications^d^	6 (14.3%)	9 (21.4%)	0.569	0.7 (0.3–1.7)
Post dural puncture headache	2 (4.8%)	5 (11.9%)	0.430	0.4 (0.1–1.9)
Postpartum back pain	5 (11.9%)	5 (11.9%)	> 0.999	1 (0.3–3.2)
Epidural blood patch	0 (0%)	1 (2.4%)	1.000	NA
Patient satisfaction with overall labor analgesia^e^	10 [9–10]	10 [8–10]	0.472	0 (− 1 to 1)

Values are median [IQR] or No. (%). *CSE* combined spinal-epidural, *NRS* numerical rating scale. NA indicates non-estimable due to zero frequency.

^a^Labor pain was asked after the completion of CSE procedure.

^b^Average labor pain score after initiation of CSE analgesia until delivery was specifically asked following the delivery of baby.

^c^Failure of labor analgesia was defined as the need to reinsert a new epidural catheter owing to lack of sufficient analgesia within 2 h of the primary insertion.

^d^Periprocedural composite complications were defined as radicular pain or paresthesia or bloody tap or accidental dural puncture with epidural needle during the combined spinal-epidural procedure.

^e^Patient satisfaction score with overall labor analgesia was asked following the delivery of baby using 11-point scales (0 = very unsatisfied, 10 = very satisfied).

In the ultrasound group, the mean difference between the depth measured using handheld ultrasound and the actual epidural needle depth at LOR was − 0.22 cm (95% upper limit of agreement, 0.48 cm; 95% lower limit of agreement, − 0.93 cm; Supplemental Fig. S1). Additionally, a Bland–Altman plot of the agreement between the depth measured by ultrasound and the actual spinal needle depth at dural puncture is presented in Supplemental Fig. S2.

## Discussion

In this randomized controlled trial, we found no significant difference in procedural time for CSE analgesia between the handheld ultrasound-assisted and landmark-guided techniques. However, the use of handheld ultrasound demonstrated better results in terms of the performance time, the epidural success rate at the first puncture attempt, and the number of insertion attempts.

To our best knowledge, this is the first study to compare handheld ultrasound assistance with conventional landmark palpation for CSE analgesia in laboring women. The use of handheld ultrasound can be beneficial in the clinical setting of labor CSE analgesia, which is typically performed on demand at a maternity unit separate from the main operating theater. The time and effort required for ultrasound preparation can be minimized by using a portable handheld device instead of a console machine. Furthermore, the Accuro device does not require much expertise compared to a laptop or console-type ultrasound, as it is equipped with an algorithm that automatically recognizes neuraxial structures. Our results suggested that anesthetic providers might consider using handheld ultrasound in labor CSE analgesia owing to its equivalent total time but a shorter performance time and a smaller number of puncture attempts.

We chose the procedural time as the primary outcome of this study because we considered that it could be one of the most important determinant of the first choice for neuraxial technique in clinical practice^16^. Rapid onset of sacral analgesia in CSE analgesia is more advantageous for mothers in the later stages of labor or with the rapid progress of labor^19^; and in these parturients, rapid completion of the procedure is desirable. Previously, the preprocedural ultrasound has been suggested to prolong the total procedure time compared with landmark palpation^13,16,20,21^. However, a recent systematic review found no evidence of a difference in the total time taken to perform neuraxial anesthesia between the two techniques in obstetrics^13^. Ghisi et al. reported that the total procedure time was longer when using handheld ultrasound compared with landmark palpation for spinal anesthesia in orthopedic patients^22^. However, they applied a sterile cover on the handheld ultrasound device, which may account for the longer procedure time in the ultrasound group^22^. In the current study, we purposely excluded the additional time for sterile drape and local anesthetic infiltration from the primary outcome because it is irrelevant to the technique itself. Our data indicated that practitioners could use preprocedural handheld ultrasound for CSE analgesia without concerns about prolonging the procedure time.

Failure to obtain CSF on the first spinal attempt during the NTN technique may be attributed to incorrect placement or excessive paramedian deviation of the epidural needle^4,9,23,24^. Therefore, the midline should be accurately located during epidural needle placement to avoid failure of the spinal component of the CSE technique. Preprocedural ultrasound has been suggested to locate the midline accurately^4^. Tao et al. found that using ultrasound reduced the procedural time of the spinal component of CSE anesthesia in patients undergoing cesarean delivery^4^. Similarly, our results showed decrease in time from the first LOR until CSF efflux in the ultrasound group. However, the number of redirections required to obtain the CSF efflux showed no significant intergroup difference.

A high correlation between the depth measured by console ultrasound and actual needle depth was reported^10,25–27^. The depth measured by handheld ultrasound also successfully predicted the actual needle depth^28^, even when compared with the console ultrasound device^29^. Carvalho et al. reported that the Accuro and console ultrasounds provided comparable epidural depth estimates and allowed anesthesiologists to anticipate the LOR within 0.8 cm^29^. Our data indicate that an anesthetic provider could anticipate the depth of LOR within the depth measured by the Accuro device + 0.93 cm.

Theoretically, the use of preprocedural ultrasound has potential to reduce the complications of neuraxial anesthesia^10^. Several studies have reported that the use of ultrasound reduced the risk of traumatic procedures^14,30^. The risk of an accidental dural puncture can be potentially reduced by the ability to measure the epidural depth, as anesthesiologists can perform more focused procedures near the anticipated depth^30^. In our study, accidental dural puncture occurred in none of the patients in the ultrasound group versus five patients in the palpation group; however, the difference was not statistically significant. Surprisingly, in the palpation group, unintentional dural puncture occurred at a higher rate than the previously reported incidence of 1–3% in obstetric patients^31,32^, as well as what we have experienced in our institution. These results were unexpected, considering that all CSE procedures were conducted by experienced anesthesiologists, and the reasons for these occurrences are not clear. It might be partially explained by the observation that, in five patients where accidental dural puncture occurred, the distance between the epidural depth at which loss of resistance and dural puncture was very small. Given the high incidence of unintentional dural puncture in the palpation group, care should be taken when interpreting our results. We observed no intergroup differences in post-dural puncture headaches or epidural blood patches. However, this study was not sufficiently powered to detect differences in the incidence of complications between the two techniques. Future investigations with larger sample sizes are required to determine the effect of ultrasound use on the safety profile of CSE anesthesia.

Our study had limitations. First, we could not blind the performer to the group allocations because of the nature of the study. Further studies using blinded techniques are required. Second, this study was conducted in healthy pregnant women with relatively low body mass index. Although we performed an exploratory subgroup analysis of mothers with a body mass index > 30 mg/m^2^, it was not sufficiently powered. Since the clinical usefulness of handheld ultrasound may be more prominent in mothers with anticipated technical difficulties, future studies are required in mothers with predictors of technical difficulty. Third, experienced anesthesiologists performed the procedures. Further trials with procedures performed by novices or resident anesthesiologists are required. Fourth, we determined the sample size based on the assumption that a difference of > 1 min in the procedural time between the groups would be clinically significant for patient comfort and satisfaction. It would be uncomfortable for mothers to maintain a rather strenuous position for CSE while experiencing ongoing labor pain. However, it should be noted that a 1-min difference is unlikely to be associated with significant clinical adverse events. Finally, this study did not compare the efficacy between the handheld versus standard ultrasound. Future studies are needed to investigate the comparative efficacy of the handheld and standard ultrasound-assistance for labor CSE analgesia.

In conclusion, we observed no significant difference in the total procedure time between handheld ultrasound assistance and landmark palpation in the CSE technique in mothers receiving labor analgesia. However, the use of preprocedural handheld ultrasound resulted in a reduction in the performance time and an improvement in the first-attempt success rate. These findings indicate that handheld ultrasound may be a valuable option for labor CSE analgesia. Future studies in mothers with predictors of technical difficulty with procedures performed by inexperienced anesthesiologists, such as trainees and resident anesthesiologists, are required.

## Methods

### Trial design and patients

This prospective randomized controlled study was conducted at Seoul National University Hospital, Seoul, Republic of Korea, from March 5, 2021 to December 23, 2021. This study was approved by the Institutional Review Board of Seoul National University Hospital (reference number 2101-016-1186) and registered at https://clinicaltrials.gov/ct2/show/NCT04759547 (ClinicalTrials.gov Identifier NCT04759547, date of registration: February 18, 2021). All participants provided written informed consent prior to participation. This manuscript adheres to the Consolidated Standards of Reporting Trials (CONSORT) statement^33^. All study procedures were conducted in accordance with the Declaration of Helsinki. Eligible patients were healthy, term, laboring pregnant women who requested neuraxial analgesia with 2–5 cm cervical dilation. Patients with contraindications for neuraxial anesthesia (hypersensitivity to local anesthetics, coagulopathy, or local infection around the puncture site), severe cardiovascular disease, history of lumbar spinal surgery, anatomical abnormalities of the lumbar spine, and of age < 18 years were excluded.

### Randomization

Patients were randomly assigned to either the preprocedural handheld ultrasound-assisted technique group (ultrasound group) or the conventional palpation-guided technique group (palpation group) using computer-generated random numbers secured in sealed, opaque envelopes. The CSE procedures were performed by one of the three anesthesiologists (S-KP, JB, and YK); each anesthesiologist had performed over 50 neuraxial ultrasound scans prior to this study. Block randomization was conducted to balance the allocation of the anesthesiologists to each group.

### Study intervention

Labor CSE analgesia was performed with the patient in the lateral decubitus position. In the ultrasound group, a wireless handheld ultrasound device (Accuro, Rivanna Medical, Charlottesville, VA, USA) equipped with pattern recognition software was used to identify bony landmarks and estimate the depth of the epidural space. Before skin disinfection, a preprocedural examination was done using the handheld ultrasound at the L4/5 and L3/4 levels, as described previously^22,28,29^. Briefly, the midline was identified by sliding the probe laterally until the dashed indicator turned orange. The probe was then moved longitudinally along the midline until the integrated software detected the interspace (Supplemental Fig. S3A). A device locator was used to mark the skin, and the intersection of the four cutaneous marks was used as the needle entry point (Supplemental Fig. S3B). After skin disinfection, CSE anesthesia was administered based on the skin markings. In the palpation group, the spinous process and iliac crest were palpated to find the L4/5 and L3/4 interspaces before and after disinfection. CSE anesthesia was performed at an appropriate location determined by palpation of the surface landmarks.

**Figure 2:**
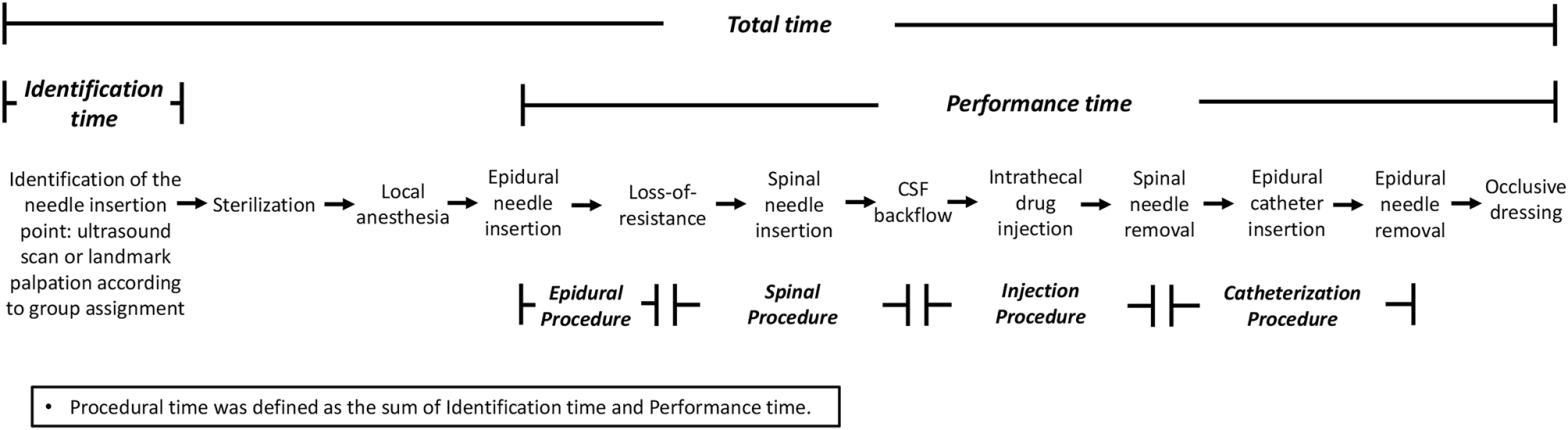
The diagram of the combined spinal-epidural analgesia procedure. *CSF* cerebrospinal fluid.

In both groups, lidocaine skin infiltration was performed at the planned puncture site. A schematic of the CSE procedure is shown in Fig. 2. The epidural space was located using a standard loss-of-resistance (LOR) to air technique with a CSE needle (CSEcure, Portex Combined Spinal/Epidural minipack 27G/18G, ICU Medical, Inc., San Clemente, CA). After locating the epidural space, a 27G pencil-point spinal needle was inserted through the epidural needle using the NTN technique. Bupivacaine 1.5 mg (0.5% bupivacaine, 0.3 mL) and fentanyl 15 μg were intrathecally injected after confirming cerebrospinal fluid (CSF) efflux, and the spinal needle was removed. Subsequently, an epidural catheter was inserted 4 cm into the epidural space. If there was no CSF return after adjusting the depth of the spinal needle, the process of locating the epidural space was repeated by redirecting the epidural needle. If the epidural space was not found after five separate insertion attempts, the anesthesiologist could use alternative methods (ultrasound group: landmark palpation, console ultrasound, or paramedian approach; palpation group: handheld or console ultrasound, or paramedian approach).

The anesthesiologist formally graded the ease of landmark palpation on a 4-point scale (easy, moderate, difficult, or impossible) after the completion of the procedure to ensure that the procedural time included only the time essential to the CSE procedure in both groups^34–36^. After placing the patient in a supine position, 3 mL of 0.15% ropivacaine was injected into the epidural catheter following aspiration. An epidural solution of 0.1% ropivacaine with fentanyl (2 μg/mL) was infused via a programmed intermittent epidural bolus pump (Accumate 1200 electronic infusion pump, Woo Young Medical, Seoul, Korea) for maintenance of analgesia. All patients received a programmed bolus of 6 mL every 45 min, with patient-controlled epidural boluses of 4 mL with a 15-min lockout time, following our institution’s practice.

### Outcomes

Primary outcome was the procedural time of CSE anesthesia, defined as the sum of the identification time and performance time (Fig. 2). The identification time was defined as the time from the placement of ultrasound on the patient’s skin to the anesthesiologist’s declaration of completion of skin marking in the ultrasound group and the time from the first touch for palpation to completion of palpation in the palpation group. The performance time was defined as the time from epidural needle insertion to the end of application of the occlusive dressing of the epidural catheter. We excluded the time taken for sterile drape or lidocaine skin infiltration from the primary outcome because it was considered as irrelevant to the technique itself.

The secondary outcomes were the epidural success rate (overall, at the first skin puncture attempt, and at the first needle pass without redirections), success rate of dural puncture through the NTN technique (overall and at the first spinal needle pass without redirections), number of needle passes (defined as the number of needle redirections without removing the needle from the skin, including the first pass) for the first successful epidural placement, number of epidural needle insertion attempts (defined as the number of each needle punctures through the skin) for the successful CSE placement, number of epidural needle redirections for success of NTN technique, use of alternative methods, number of interspace levels at which insertion was attempted, depth to the ligamentum flavum-dura mater complex measured by handheld ultrasound, actual needle depth at LOR, and actual spinal needle depth at CSF return. We measured the spinal needle depth using the scale marked on the CSE needle (Portex CSEcure). We also assessed periprocedural complications, including radicular pain, paresthesia, bloody tapping, inadvertent dural puncture with the epidural needle, failure of labor analgesia (defined as the need to reinsert a new epidural catheter owing to lack of sufficient analgesia within 2 h of the primary insertion), post-dural puncture headache, and incidence of back pain at the site of epidural insertion. Labor pain before and after analgesia (11-point numeric scale, 0 = no pain, 10 = worst pain imaginable), back pain during the procedure (0 = no pain, 10 = worst pain imaginable), discomfort during the procedure (0 = no discomfort, 10 = most discomfort imaginable), and patient satisfaction score with overall labor analgesia (0 = very unsatisfied, 10 = very satisfied) were also evaluated.

The successful epidural placement was confirmed by the LOR to air, and the successful spinal placement was defined as the presence of CSF return with the spinal needle placed through the epidural needle. The time of each component of the CSE procedure was also recorded as follows (Fig. 2): time from Tuohy needle insertion to the first LOR, time from the LOR to CSF efflux, time from CSF efflux to the removal of the spinal needle after drug injection, and time from the removal of the spinal needle to the removal of the Tuohy needle after catheter insertion. The total time including preparation time, defined as the time from the start of identification using ultrasound or palpation to the end of application of the dressing, was recorded.

Two independent observers recorded the outcomes. The intra-procedural outcome assessor could not be blinded to the group allocation owing to the presence of skin markings in the ultrasound group. However, a blinded observer entered the room after completing the CSE procedure and evaluated the post-procedural outcomes. An electronic timer was used to accurately record each time interval. Independent research staff pressed the lap button of the stopwatch at each time point and recorded the time intervals.

### Statistical analysis

Based on a previous study, the mean procedural time of CSE analgesia using a conventional landmark-guided technique was assumed to be 7.67 (standard deviation 1.52) min^21^. We assumed that a difference of > 1 min in the procedural time between the groups would be clinically significant. With a power of 80% and a two-sided significance level of 0.05, a sample size of 38 per group was required. Accounting for a drop-out rate of 10%, we determined a sample size of 42 per group.

All analyses were performed on an intention-to-treat basis. Continuous variables were tested for normality using Q–Q plots. Normally distributed data were analyzed using Student’s *t*-test. For nonnormally distributed data, the Mann–Whitney *U* test was used to investigate the differences between the groups, and confidence intervals (CI) for the median differences were calculated using the wilcox.test function in R software. Categorial variables were analyzed using the χ^2^ test or Fisher’s exact test, as appropriate. The relative risk of binary variables was presented with 95% CI. The agreement between epidural depth measured by handheld ultrasound and actual needle depth was calculated using Bland–Altman analysis, and the upper and lower 95% limits of agreement were determined. We conducted an exploratory subgroup analysis on mothers with a body mass index > 30 kg/m^2^. Statistical significance was set at *P* < 0.05. Analyses were performed using SPSS (version 25; IBM corp., Armonk, NY) and R (version 4.1.0; R Foundation for Statistical Computing, Vienna, Austria).

### Supplementary Information

Supplementary Information.

## Data Availability

The datasets are available from the corresponding author on reasonable request.
