# Supplementary material for: Handheld ultrasound-assisted versus palpation-guided combined spinal-epidural for labor analgesia: a randomized controlled trial
Source: Sci Rep. 2023 Dec 27;13:23009. doi: 10.1038/s41598-023-50407-7 (PMC10754906; doi:10.1038/s41598-023-50407-7)
Supplement: Supplementary file 1 — Supplementary Information. [file 41598_2023_50407_MOESM1_ESM.docx]

**Supplemental Digital Content**

**Handheld ultrasound-assisted versus palpation-guided combined spinal-epidural for labor analgesia: a randomized controlled trial**

**List of Supplemental Materials**

**Supplemental Table S1.** Analysis of identifying and procedural time and number of needle passes/attempts by anesthesiologists who performed combined spinal-epidural anesthesia.

**Supplemental Table S2.** Subgroup analysis of parturients with a body mass index > 30 kg/m^2^.

**Supplemental Figure S1.** Bland-Altman plot of the agreement between the epidural depth measured using handheld ultrasound and the actual epidural needle depth at loss of resistance. The y-axis represents the difference between the measured depth and the actual needle depth, and the x-axis represents the average. Mean bias with 95% limits of agreement are presented. UD, depth measured using handheld ultrasound; ND, actual epidural needle depth.

**Supplemental Figure S2.** Bland-Altman plot of the agreement between the depth measured using handheld ultrasound and the actual spinal needle depth at dural puncture. The y-axis represents the difference between the measured depth and the actual needle depth, and the x-axis represents the average. Mean bias with 95% limits of agreement are presented. UD, depth measured using handheld ultrasound; SND, actual spinal needle depth.

**Supplemental Figure S3.** Preprocedural handheld ultrasound examination for combined spinal-epidural analgesia. A, A scan was performed with the patient in the lateral decubitus position. The handheld ultrasound screen displays the identified interspinous space and the estimated epidural depth. B, Once the desired interspinous space was identified, the skin was marked by gently pressing the device locator against the skin. The midpoint of the probe was used as the needle insertion point.

**Supplemental Table S1**. Analysis of identifying and procedural time and number of needle passes/attempts by anesthesiologists who performed combined spinal-epidural anesthesia.

| Both groups |  | Anesthesiologist A (n=28) | Anesthesiologist B (n=28) | Anesthesiologist C (n=28) | *P* value |
| --- | --- | --- | --- | --- | --- |
|  | Identifying and procedural time, seconds | 194 (154.5–251.5 [135–1260]) | 182.5 (163.5–318.5 [116–562]) | 236.5 (184–325.5 [115–598]) | 0.276 |
|  | Number of passes | 2 (1–4 [1–15]) | 2 (1–4 [1–11]) | 2 (1–3.5 [1–15]) | 0.729 |
|  | Number of attempts | 1 (1–1.5 [1–4]) | 1 (1–1.5 [1–4]) | 1 (1–2 [1–4]) | 0.747 |
| Ultrasound group |  | Anesthesiologist A (n=14) | Anesthesiologist B (n=14) | Anesthesiologist C (n=14) |  |
|  | Identifying and procedural time, seconds | 203.5 (154–216 [135–298]) | 179 (166–224 [144–455]) | 196.5 (182–269 [156–598]) | 0.610 |
|  | Number of passes | 2 (1–2 [1–5]) | 2 (1–3 [1–7]) | 2 (1–3 [1–15]) | 0.643 |
|  | Number of attempts | 1 (1–1 [1–2]) | 1 (1–1 [1–4]) | 1 (1–1 [1–4]) | 0.536 |
| Palpation group |  | Anesthesiologist A (n=14) | Anesthesiologist B (n=14) | Anesthesiologist C (n=14) |  |
|  | Identifying and procedural time, seconds | 183 (155–370 [138–1260]) | 189.5 (161–354 [116–562]) | 271 (186–359 [115–520]) | 0.582 |
|  | Number of passes | 3 (1–6 [1–15]) | 1.5 (1–5 [1–11]) | 3 (1–6 [1–12]) | 0.743 |
|  | Number of attempts | 1 (1–2 [1–4]) | 1 (1–2 [1–3]) | 1.5 (1–2 [1–3]) | 0.562 |

Values are median (IQR [range]).

*P* values are the results of Kruskal–Wallis test.

**Supplemental Table S2.** Subgroup analysis of parturients with a body mass index > 30 kg/m^2^.

|  | Ultrasound group (n=9) | Palpation group (n=5) | *P* value |
| --- | --- | --- | --- |
| Body-mass index (kg/m^2^) | 31.2 (31.2–34.0) | 32.2 (31.0–32.9) | 1.000 |
| Procedure time (s)*^a^* | 298 (199–455) | 359 (283–423) | 0.438 |
| Identification time (s)*^b^* | 47 (44–53) | 52 (42–60) | 1.000 |
| Performance time (s)*^c^* | 176 (155–204) | 299 (243–381) | 0.042 |
| Epidural success rate |  |  |  |
| Overall | 9 (100%) | 5 (100%) |  |
| At first skin puncture attempt | 8 (88.9%) | 1 (20%) | 0.046 |
| At first needle pass | 2 (22.2%) | 0 (0%) | 0.733 |
| Needle-through-needle spinal success rate |  |  |  |
| Overall | 9 (100%) | 4 (80%) | 0.757 |
| At first spinal needle pass | 7 (77.8%) | 3 (60%) | 0.930 |
| Number of epidural needle insertion attempts^d^ | 1 (1–1) | 2 (2–2) | 0.035 |
| Number of needle passes^e^ | 2 (2–3) | 7 (3–8) | 0.020 |
| Periprocedural pain score (NRS) | 3 (1–4) | 4.5 (3–5) | 0.201 |
| Periprocedural patient discomfort score (NRS) | 2 (1–3) | 6 (5–7) | 0.044 |

Values are median (IQR) or number (percentage). NRS, numeric rating scale.

*^a^* Procedure time was defined as the sum of identification time and performance time.

*^b^* Identification time was defined as the time from the placement of ultrasound on the patient’s skin and the anesthesiologist’s declaration of completion of skin marking in the ultrasound group and the time from the first touch for palpation to completion of palpation in the palpation group.

*^c^* Performance time was defined as the time from epidural needle insertion to the end of application of the occlusive dressing of the epidural catheter.

^d^ Number of epidural needle insertion attempts was defined as the number of individual needle skin punctures until the successful combined spinal-epidural placement.

^e^ Number of needle passes was defined as the number of needle redirections without removing the needle from the skin until the first successful epidural placement.


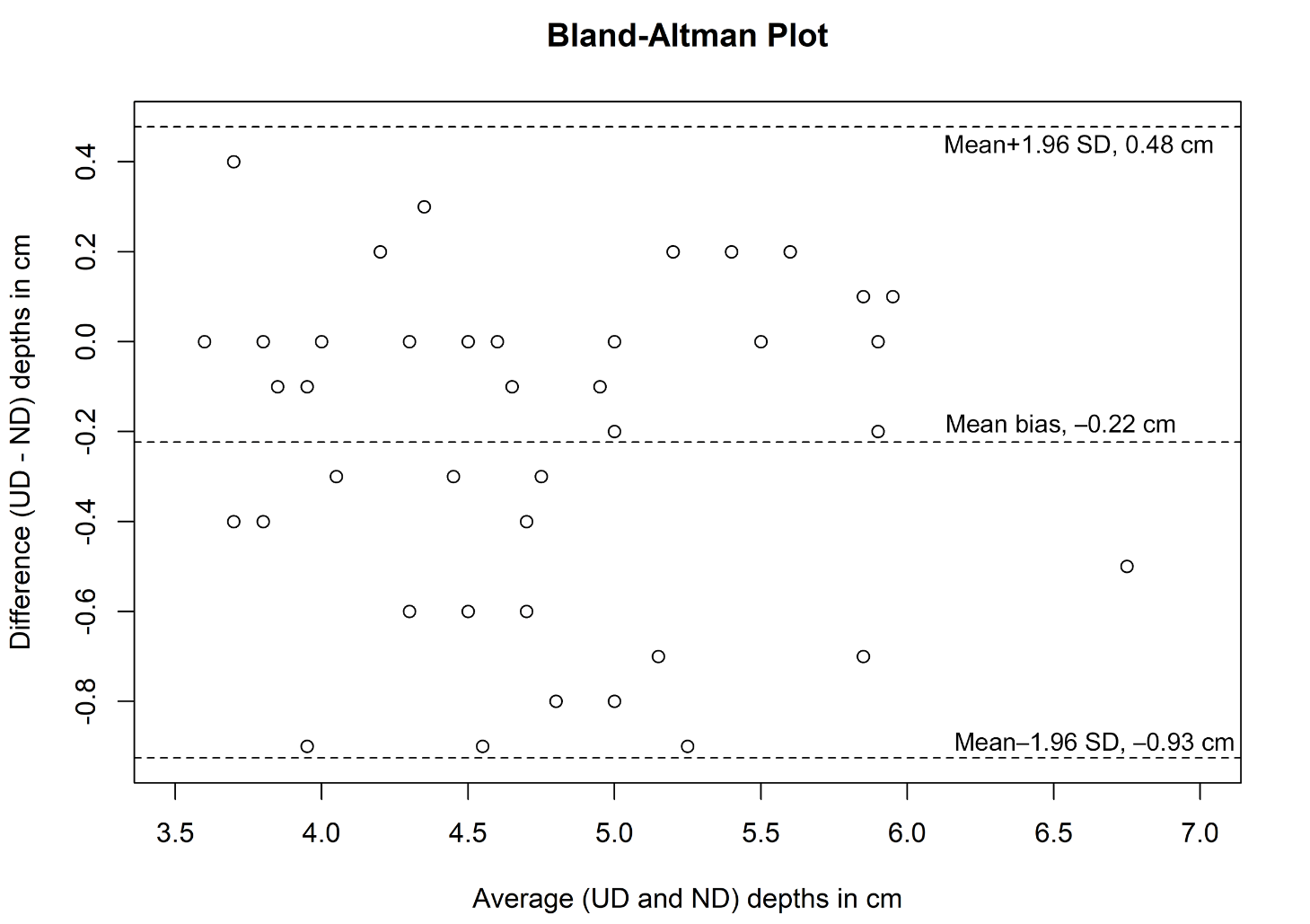


**Supplemental Figure S1.** Bland-Altman plot of the agreement between the epidural depth measured using handheld ultrasound and the actual epidural needle depth at loss of resistance. The y-axis represents the difference between the measured depth and the actual needle depth, and the x-axis represents the average. Mean bias with 95% limits of agreement are presented. UD, depth measured using handheld ultrasound; ND, actual epidural needle depth.


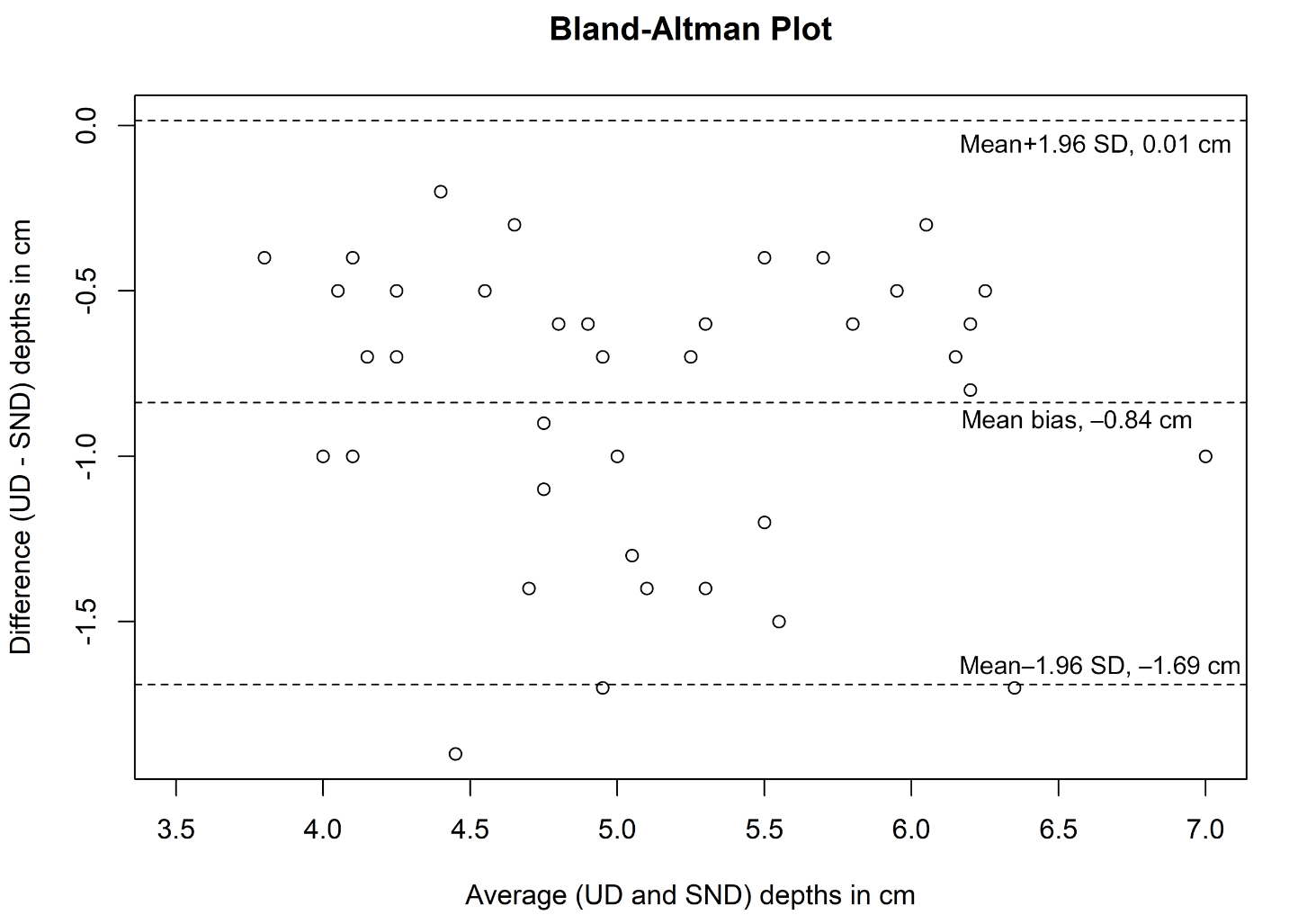


**Supplemental Figure S2.** Bland-Altman plot of the agreement between the depth measured using handheld ultrasound and the actual spinal needle depth at dural puncture. The y-axis represents the difference between the measured depth and the actual needle depth, and the x-axis represents the average. Mean bias with 95% limits of agreement are presented. UD, depth measured using handheld ultrasound; SND, actual spinal needle depth.


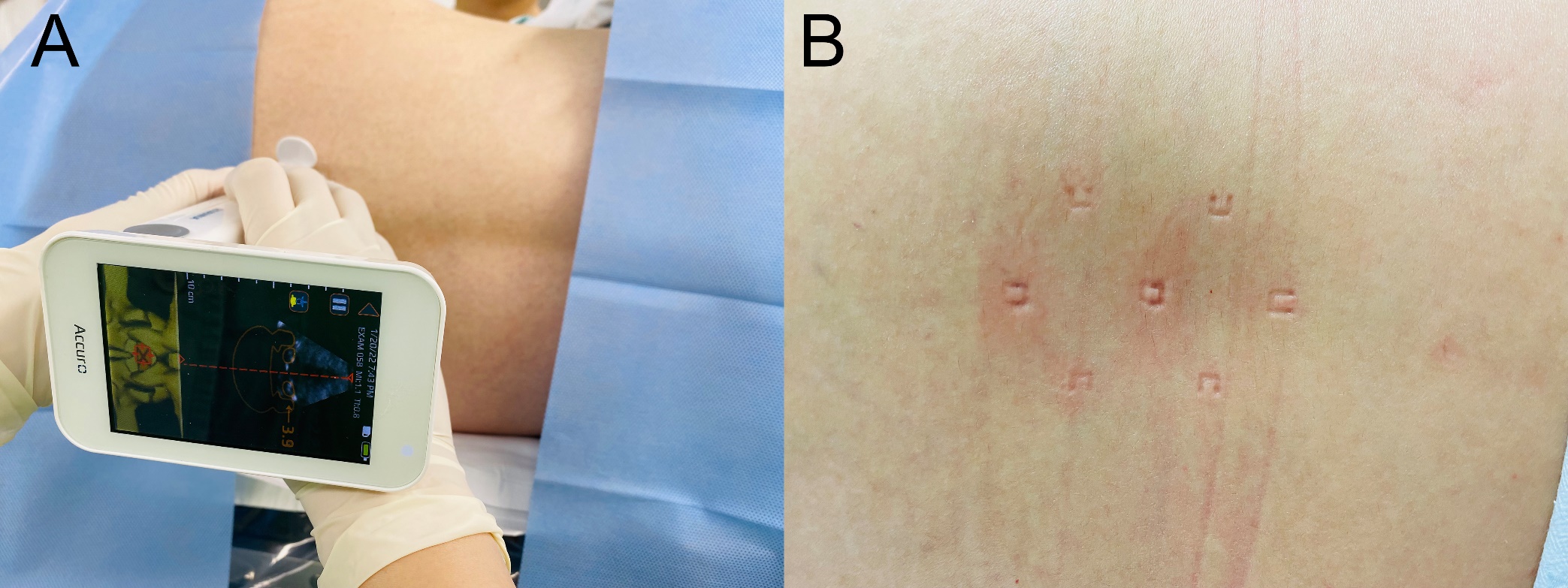


**Supplemental Figure S3.** Preprocedural handheld ultrasound examination for combined spinal-epidural analgesia. A, A scan was performed with the patient in the lateral decubitus position. The handheld ultrasound screen displays the identified interspinous space and the estimated epidural depth. B, Once the desired interspinous space was identified, the skin was marked by gently pressing the device locator against the skin. The midpoint of the probe was used as the needle insertion point.
